# Effects of low crude protein and high-fiber diets on production performance, egg quality, behavior, and integument condition in laying hens

**DOI:** 10.1016/j.psj.2026.107369

**Published:** 2026-07-01

**Authors:** Muhammad Adnan Aslam, Helena Wall, Anette Wichman, Emma Ivarsson

**Affiliations:** Department of Applied Animal Science and Welfare, Swedish University of Agricultural Sciences (SLU), Uppsala, Sweden

**Keywords:** Laying hen, Low crude protein, Insoluble fiber, Integument condition, Egg quality

## Abstract

Low crude protein (LCP) diets supplemented with essential amino acids may reduce the environmental impact of poultry production, whereas high-fiber diets containing increased insoluble non-starch polysaccharides (iNSP) may improve hen welfare by reducing feather and injurious pecking. To investigate the effects of these dietary strategies on laying hens, a total of 1,600 sixteen-week-old Bovans Robust White hens were randomly assigned to 16 pens and fed experimental diets from 16 to 50 weeks of age in a 2 × 2 factorial arrangement with two crude protein levels (standard, SCP; low, LCP) and two iNSP levels (standard fiber, SF; high fiber, HF). The LCP diets contained 1.5% units less crude protein, and HF diets contained 2% units more iNSP (via oat hull inclusion) than their respective standard diets. All diets were formulated to contain similar levels of digestible amino acids. Feed intake, egg production, mortality, egg quality, and integument condition were recorded. Fearfulness was assessed through a novel object test, and pecking behavior was evaluated by direct observation. Data were analyzed using linear mixed-effects models in R. Feed intake was significantly lower in the HF group than the SF group from 32 to 43 weeks of age (P < 0.001). Fiber level did not affect body weight at 34 weeks of age; however, at 49 weeks, hens in the HF group had higher body weight than those in the SF group (P = 0.003). Hens fed HF diets exhibited improved feather cover (P = 0.016), fewer rear body injuries (P = 0.012), higher egg weight (P = 0.010), and increased egg mass (P = 0.011) compared to hens fed SF diets. LCP diets resulted in lower egg weight (P = 0.015) and higher yolk color (P < 0.001) than SCP diets. Dietary treatments did not affect lay percentage, mortality, novel object responses, eggshell thickness, eggshell weight, yolk weight, or albumen dry matter. In conclusion, LCP diets supplemented with essential amino acids maintained lay percentage, and HF diets improved plumage condition and reduced rear body pecking injuries among laying hens.

## Introduction

With the increasing global population, the demand for eggs is rising worldwide. At the same time, consumer concern for animal welfare and sustainability is increasingly influencing egg production systems (Gautron et al., 2021). Protein is both an essential and expensive component of poultry feed. Traditionally, laying hens have been fed higher levels of protein than what may be needed to ensure an adequate supply of amino acids for optimal performance (Chathuranga and Heo, 2025). However, in recent years, interest in low crude protein (LCP) diets has grown substantially because of their ability to reduce the environmental impact from poultry production by decreasing nitrogen excretion and in turn ammonia emission (Liu et al., 2025). Beyond reducing pollution, LCP diets also offer the potential to decrease the dependence on imported soybeans and reduce feed costs in regions where high-quality protein sources are not readily available (Harn et al., 2021). Notably, earlier studies have shown that LCP diets can maintain production performance when supplemented with amino acids (Keshavarz and Austic, 2004; Burley et al., 2013; Parenteau et al., 2020). Adequate levels of essential amino acids, for instance, Methionine, Lysine, Threonine, Tryptophan, Valine, Arginine, and Isoleucine are crucial for enabling laying hens to achieve optimal growth and production (Bregendahl et al., 2008). A LCP diet deficient in amino acids can result in poor production performance, reduced egg quality, and an increased risk of poor feather cover due to feather pecking (Emous and Krimpen, 2019; Geng et al., 2021).

Commercial laying hens are typically fed diets containing approximately 17.5 to 18.5% crude protein during the early to mid-lay period (Bovans, 2015). Marked reductions in dietary crude protein, for example from 18% to 13.5% or 12%, have been reported to impair production performance, even when supplemented with amino acids (Harn et al., 2021). However, several studies have shown that reducing dietary crude protein by around 2% units when supplemented with essential amino acids does not adversely impact egg production, body weight, egg weight, or feed efficiency (Rama Rao et al., 2011; Ji et al., 2014; Parenteau et al., 2020). On the other hand, some studies have reported an increased feed intake with LCP diets supplemented with amino acids (Mousavi et al., 2013; Harn et al., 2021). Moreover, others have found a negative effect on feather condition due to increased feather pecking caused by deficiencies or imbalances of essential amino acids, which is more likely to occur among hens fed LCP diets (Ambrosen and Petersen, 1997).

Dietary fiber can have several positive effects for laying hens, such as enhanced gut development (Sacranie et al., 2012; Yokhana et al., 2016; Röhe et al., 2019; Sozcu and Ipek, 2020), a positive shift in gut microbiota, reduced feather pecking (Bearse et al., 1940; Kalmendal and Wall, 2012; Qaisrani et al., 2013), and improved production performance (Incharoen and Maneechote, 2013; Sozcu and Ipek, 2020; Koçer et al., 2021). High-fiber diets can result in higher nitrogen retention within the body (Roberts et al., 2007a). Additionally, a high-fiber diet can reduce the environmental impact of egg production by shifting nitrogen excretion from readily degradable uric acid to more stable microbial protein in feces (Tetens et al., 1996), which is less likely to degrade into ammonia (Roberts et al., 2007a). Oat hulls are widely recognized as a rich source of insoluble fiber, and their positive impact on the prevention of feather pecking and cannibalism is well established (Bearse et al., 1940). Some recent studies have also reported beneficial effects of high-fiber diets supplemented with oat hulls on feather condition (van Krimpen et al., 2009; Qaisrani et al., 2013; Patt et al., 2022).

Diet has a direct impact on hen health and production, and it can also indirectly influence the behavior of laying hens (Herwig et al., 2019). A high-fiber or LCP diet may modulate fearfulness, as high-fiber diets can improve satiety and reduce feather pecking, potentially lowering fear responses, whereas previously reported negative effects of LCP on feather condition may increase fearfulness (Uitdehaag et al., 2008; Rodenburg et al., 2013; Hüttner et al., 2023).

Although considerable research has evaluated LCP and high-fiber diets separately, relatively few studies have examined LCP diets combined with elevated fiber levels, and these studies were conducted in cage systems housing small groups of layers (Roberts et al., 2007a; 2007b; Praes et al., 2011, 2014) . To the best of our knowledge, no study has investigated the combined effects of LCP and higher fiber levels on integument condition and behavior in laying hens. With the ban on the conventional battery cage systems in the EU (Council Directive, 1999*/74/EC*) and increasing consumer concern regarding animal welfare, alternative non-cage production systems, such as floor and aviary systems, are gaining prominence (Majewski et al., 2024). Eits et al. (2005) reported that the housing system can influence dietary protein requirements, with floor-housed hens requiring a lower crude protein concentration (% CP in the diet) than caged hens. This may be related to the higher activity levels of floor-housed hens, which increase energy requirements and may consequently stimulate greater feed intake. However, high-fiber diets may reduce voluntary feed intake by increasing digesta retention time in the crop and foregut (Hetland et al., 2004). Therefore, studies evaluating the combined effects of LCP and high-fiber diets in laying hens housed in larger groups are needed.

The present study evaluated the effects of diets formulated with standard or LCP levels in combination with standard or high fiber levels on production performance, integument condition, egg quality, and behavior in laying hens. We hypothesize that: 1) A lower crude protein diet, supplemented with essential amino acids, will sustain egg production, egg quality, feed intake, and feed conversion ratios at levels comparable to those of standard diets. 2) A higher inclusion of fiber will reduce feather pecking, injurious pecking, and fearfulness.

## Materials and methods

### Animals and housing

**Fig. 1:**
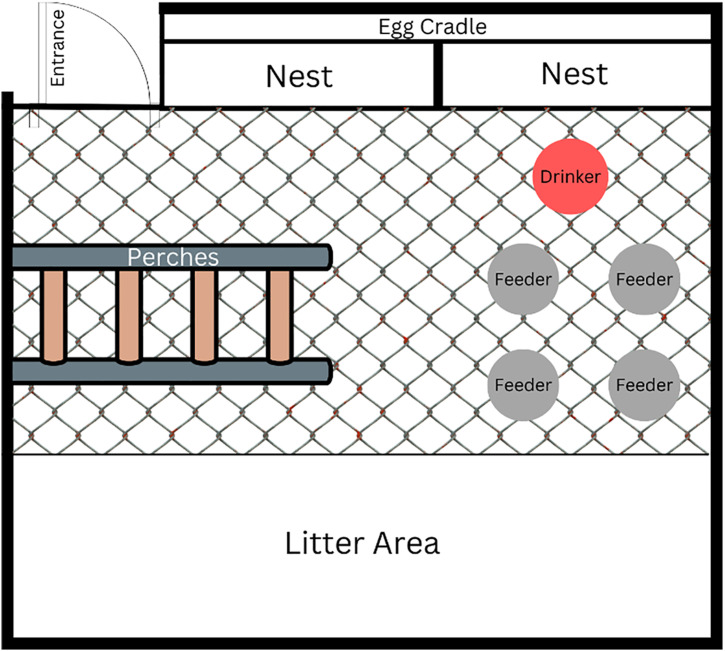
Schematic layout of an experimental pen. Each pen (n = 16) consisted of a littered area, a slatted area, and nests. Feeders, drinkers, and perches were located in the slatted area.

This experiment was conducted on 1600 Bovans Robust White laying hens that were obtained from a commercial rearing farm (Närkesberg Hönseri AB, Åsbro, Sweden) at 16 weeks of age. All experimental procedures were approved by the Animal Ethics Committee of the Uppsala region in Sweden (Decision number 5.8.18-20113/2022). The trial was carried out at the Swedish Livestock Research Centre, Lövsta for a total of 34 weeks until the hens reached 50 weeks of age. The hens were non-beak trimmed and vaccinated against infectious bronchitis virus, Marek’s disease, coccidiosis, and chicken encephalomyelitis. They were housed in 16 identical pens with a single-tier floor system (Fig. 1). Each pen (3.56 × 3.56 m; W × L) housed 100 hens, corresponding to a stocking density of 7.9 hens/m² based on total floor area. Each pen possessed a raised slatted floor area (2.30 m × 3.56 m, W × L), perches, and a litter area (1.32 m × 3.56 m, W × L) with wood shavings. An automatic floor scraper was used to remove the manure below the slatted area. Additionally, each pen contained two colony nest boxes, each measuring 46 cm × 115 cm, placed on the slatted floor. The hens had continuous access to feed from four feeders and water which was provided through one bell drinker. They were provided with 9 h of light per day at 16 weeks of age, which gradually increased to 15 h by 22 weeks of age, and was thereafter maintained. An automatic ventilation and heating system was used to maintain the temperature between 20 and 26 °C. From 30 weeks of age, the hens were provided with environmental enrichment in the form of pecking materials and plastic balls. Each pen received one consumable mineral-based pecking stone (Peckstone, 30 cm diameter; Svenska Foder AB, Lidköping, Sweden), composed primarily of calcium, phosphorus, sodium, and magnesium, and one non-destructible Peck Block made of a lightweight expanded clay aggregate (LECA block; 15 × 19 × 59 cm, height × width × length; Hallsjö Brädgård AB, Uppsala, Sweden). Three perforated plastic floor balls (Innebandyboll, 7 cm diameter; Lyreco Sverige AB, Borås, Sweden) were also provided per pen. At 37 weeks of age, red mites (*Dermanyssus gallinae*) were observed in the barn and managed by administering Fluralaner (Exzolt, MSD Animal Health Sweden) in the drinking water and spraying Celite (VermiNIX™, Tergent, Sweden) following consultation with the Swedish Veterinary Agency (SVA).

### Experimental design and diet

Pens were randomly assigned to one of four dietary treatments in a 2 × 2 factorial design, with insoluble non-starch polysaccharides (iNSP) referred to as fiber (Standard Fiber vs. High Fiber) and crude protein (Standard Crude Protein vs. Low Crude Protein) as factors. This resulted in four treatments: Standard Crude Protein with Standard Fiber (SCP-SF), Standard Crude Protein with High Fiber (SCP-HF), Low Crude Protein with Standard Fiber (LCP-SF), and Low Crude Protein with High Fiber (LCP-HF), as presented in Table 1. Each treatment consisted of four replicates, with 100 hens per replicate, totaling 400 hens per treatment. The hens were fed a wheat, corn, and soybean meal-based crumbled pellet diet in two phases (Table 2). The first phase lasted until 30 weeks of age, after which phase two began. In phase one, the SCP-SF diet was optimized (Table 3) to contain 17% crude protein (CP), while the LCP-SF diet was optimized to 15.5% CP. The SCP-HF and LCP-HF diets contained the same CP levels as the SCP-SF and LCP-SF diets, respectively, but included 2% additional iNSP, which increased the fiber content from 10.7 to 12.7%. In phase two, the SCP diets were formulated to contain 16.5% CP, and the LCP diets 15.2% CP. The phase two SCP-HF and LCP-HF diets included an additional 2% fiber. All diets were formulated based on digestible amino acid values.

**Table 1:** Overview of dietary treatments in the 2 × 2 factorial design showing combinations of protein and fiber levels, and corresponding crude protein (CP) and insoluble non-starch polysaccharides (iNSP) contents in Phase 1 and Phase 2 diets.

**Treatment**^1^	**Protein Inclusion**	**Fiber Inclusion**	**Phase 1**		**Phase 2**	
			CP	iNSP	CP	iNSP
SCP-SF	Standard	Standard	17.0	10.7	16.5	10.7
SCP-HF	Standard	High	17.0	12.7	16.5	12.7
LCP-SF	Low	Standard	15.5	10.7	15.2	10.7
LCP-HF	Low	High	15.5	12.7	15.2	12.7

1 SCP-SF = Standard Crude Protein and Standard Fiber (Control Diet); SCP-HF = Standard Crude Protein and High Fiber; LCP-SF= Low Crude Protein and Standard Fiber; LCP-HF = Low Crude Protein and High Fiber.

**Table 2:** Ingredient composition of experimental diets in Phase 1 (fed from the start of the experiment until 30 weeks of age) and Phase 2 (30–50 weeks of age). Values are expressed as a percentage of diet (as-fed basis).

Feeding Phase	Phase 1				Phase 2			
Protein and Fiber Level	SCP-SF	SCP-HF	LCP-SF	LCP-HF	SCP-SF	SCP-HF	LCP-SF	LCP-HF
Ingredients composition (%)								
Wheat	41.01	32.07	33.92	25.50	41.70	31.74	38.13	33.96
Corn	10.00	10.00	20.00	19.39	10.00	10.00	14.90	10.46
Sunflower meal pellets	10.68	15.00	10.82	10.50	10.12	15.00	10.85	9.63
Limestone	9.74	9.70	9.79	9.73	10.15	10.10	10.18	10.13
Soybean meal (46% CP)	8.96	7.41	5.00	5.00	10.16	8.00	5.00	5.00
Crushed rapeseed	7.53	8.00	2.72	8.00	6.25	8.00	4.11	8.00
Feed oats	3.00	3.00	7.00	7.00	3.00	3.00	7.00	7.00
Whole oats	5.00	5.00	5.00	5.00	5.00	5.00	5.00	5.00
Oat hulls	0.00	3.94	0.00	4.56	0.00	3.78	0.00	5.49
Soybean oil	2.12	1.00	2.71	1.54	1.93	3.69	2.14	2.52
AkoFeed Soft	0.00	2.94	0.00	0.82	0.00	0.00	0.00	0.00
Monocalcium phosphate	0.49	0.48	0.53	0.53	0.29	0.27	0.32	0.34
Lysine sulfate (63%)	0.24	0.27	0.46	0.43	0.21	0.24	0.42	0.42
Bulk rock salt	0.29	0.26	0.18	0.18	0.25	0.26	0.18	0.19
Methionine	0.17	0.16	0.20	0.20	0.17	0.16	0.20	0.20
Sodium bicarbonate	0.10	0.10	0.26	0.22	0.10	0.10	0.24	0.23
Threonine	0.05	0.05	0.13	0.13	0.04	0.04	0.11	0.12
Isoleucine	0.03	0.04	0.14	0.15	0.02	0.03	0.12	0.14
Valine	0.00	0.00	0.09	0.09	0.00	0.00	0.07	0.09
Tryptophan	0.00	0.00	0.02	0.02	0.00	0.00	0.01	0.01
Premixes	0.60	0.60	1.03	1.02	0.60	0.60	1.00	1.07

SCP-SF = Standard Crude Protein and Standard Fiber (Control Diet); SCP-HF = Standard Crude Protein and High Fiber; LCP-SF= Low Crude Protein and Standard Fiber; LCP-HF = Low Crude Protein and High Fiber.

**Table 3:** Calculated nutrient composition of Phase 1 (fed from the start of the experiment until 30 weeks of age) and Phase 2 (30–50 weeks of age) diets. Values are expressed on an as-fed basis.

**Feeding Phase**		**Phase 1**				**Phase 2**			
**Protein and Fiber Level**		SCP-SF	LCP-SF	SCP-HF	LCP-HF	SCP-SF	LCP-SF	SCP-HF	LCP-HF
**Calculated nutrient composition**									
Dry Matter	%	89.0	89.1	89.6	89.6	88.9	89.0	89.4	89.5
Crude Protein	%	17.0	15.5	17.0	15.5	16.5	15.2	16.5	15.2
Crude Fat	%	7.5	6.3	9.4	8.1	7.3	6.5	9.0	8.5
Crude Fiber	%	5.4	5.2	7.0	6.8	5.2	5.2	6.4	6.7
NDF	%	13.4	13.2	16.2	16.2	134.9	137.8	159.5	167.4
Insoluble NSP	%	10.7	10.7	12.7	12.7	10.7	10.7	12.7	12.7
ME	Kcal	2700	2700	2700	2700	2700	2700	2700	2700
Phosphorus	g/kg	5.0	4.9	5.2	5.0	4.8	4.8	4.8	4.6
Calcium	g/kg	37.0	37.0	37.0	37.0	38.0	38.0	38.0	38.0
Potassium	g/kg	6.2	5.4	6.2	5.5	6.3	5.4	6.6	5.4
Sodium	g/kg	1.7	1.7	1.6	1.6	1.6	1.7	1.6	1.7
Chloride	g/kg	2.2	1.6	2.0	1.6	2.0	1.6	1.9	1.6
Linoleic Acid	g/kg	25.0	25.0	25.4	25.0	23.0	23.0	23.1	23.0
Ret P (SFR)	g/kg	3.2	3.2	3.2	3.2	3.2	3.2	3.2	3.2
Dig. Lysine	g/kg	7.7	7.7	7.7	7.7	7.5	7.5	7.5	7.5
Dig. Methionine	g/kg	4.3	4.3	4.3	4.3	4.2	4.2	4.2	4.2
Dig. Met+Cys	g/kg	7.1	6.8	7.0	6.8	6.9	6.6	6.8	6.5
Dig. Threonine	g/kg	5.4	5.4	5.4	5.4	5.3	5.3	5.3	5.3
Dig. Tryptophan	g/kg	1.9	1.7	1.8	1.7	1.8	1.7	1.8	1.7
Dig. Arginine	g/kg	10.0	9.9	10.3	9.9	9.8	9.8	9.8	9.8
Dig. Valine	g/kg	6.9	6.8	6.9	6.8	6.8	6.6	6.7	6.6
Dig. Isoleucine	g/kg	6.2	6.2	6.2	6.2	6.1	6.1	6.1	6.1
Dig. Leucine	g/kg	10.6	9.4	10.4	9.2	10.5	9.1	10.5	8.4
Dig. Histidine	g/kg	3.7	3.9	3.7	3.9	3.7	3.9	3.7	4.0
Dig. Glycine	g/kg	6.5	7.3	6.7	7.3	6.3	7.3	6.2	7.6
Dig. Serine	g/kg	6.9	5.8	6.7	5.7	6.8	5.7	6.8	5.4
Dig. Glycin+Serin	g/kg	13.4	13.1	13.4	13.0	13.1	13.0	13.0	13.0
Dig. Phenylalanine	g/kg	7.1	6.1	7.0	5.9	6.9	5.9	6.9	5.6
Dig. Tyrosine	g/kg	5.4	4.6	5.2	4.4	5.3	4.5	5.2	4.2
Dig. Alanine	g/kg	6.1	5.5	6.1	5.5	6.1	5.4	6.1	4.9
Dig. Asparagine	g/kg	12.1	9.9	12.2	10.0	12.2	9.8	12.6	9.3
Dig. Glutamine	g/kg	34.0	29.1	32.7	27.5	32.3	27.7	31.0	26.7
Dig. Proline	g/kg	10.3	9.0	9.6	8.4	9.9	8.5	9.4	8.2
Vitamin D3	IU/kg	3000	3000	3000	3000	3000	3000	3000	3000
Vitamin E	mg/kg	35	35	35	35	35	35	35	35
Vitamin A	IU/kg	10000	10000	10000	10000	10000	10000	10000	10000
Choline Chloride	mg/kg	500	500	500	500	500	500	500	500

Dig. = Digestible.

SCP-SF = Standard Crude Protein and Standard Fiber (Control Diet); SCP-HF = Standard Crude Protein and High Fiber; LCP-SF= Low Crude Protein and Standard Fiber; LCP-HF = Low Crude Protein and High Fiber.

### Experimental feed analyses

The dry matter (DM) content of the feed was assessed by oven drying at 103 °C for 16 h, followed by ashing at 550 °C for 3 h (Jennische and Larsson, 1990). Crude protein content (calculated as N × 6.25) was measured using the Kjeldahl method (NMKL, 2003), and fat content (ether extract, EE) was analyzed in accordance with the procedure outlined by the Official Journal of the European Communities (1994). Neutral detergent fiber (NDF) was analyzed according to the method described by Chai & Udén (1998). Furthermore, the amino acid composition of the feed was determined following the protocols outlined in ISO (2005). The analyzed nutrient composition is presented in Table 4.

**Table 4:** Chemical analysis of the Phase 1 and Phase 2 diets. Results are presented on an as-fed (feed sample) basis.

**Feeding Phase**		**Phase 1**				**Phase 2**			
**Protein and Fiber Level**		SCP-SF	LCP-SF	SCP-HF	LCP-HF	SCP-SF	LCP-SF	SCP-HF	LCP-HF
**Chemical analysis of experimental diets**									
Dry Matter	%	88.8	89.2	89.5	89.4	94.0	91.0	94.5	94.9
Crude Protein	%	17.3	15.7	17.6	16.0	17.7	15.9	17.6	16.1
Crude Fat	%	6.4	6.3	6.8	7.0	5.8	5.1	8.0	7.0
NDF	%	9.2	9.7	10.7	12.1	10.1	9.3	12.3	12.6
Phosphorus	g/kg	6.3	5.0	5.2	5.4	4.9	4.7	5.1	4.8
Calcium	g/kg	43.0	36.0	36.0	38.0	41.0	38.0	42.0	40.0
Potassium	g/kg	7.9	6.0	6.6	6.4	7.2	6.3	7.3	6.4
Sodium	g/kg	1.6	1.6	1.5	1.6	1.4	1.6	1.5	1.6
Magnesium	g/kg	2.7	2.1	2.2	2.2	2.3	2.2	2.4	2.1
Lysine	g/kg	8.9	8.5	9.5	8.3	8.9	9.5	9.0	9.4
Methionine	g/kg	4.6	4.5	5.0	4.5	4.8	4.7	4.7	4.6
Threonine	g/kg	6.5	6.2	7.1	6.2	6.7	6.8	6.9	6.7
Arginine	g/kg	10.8	10.2	11.4	10.3	11.4	11.0	11.3	11.3
Valine	g/kg	7.9	7.5	8.3	7.4	7.9	8.0	8.2	7.7
Isoleucine	g/kg	6.6	6.5	7.0	6.5	6.7	6.8	6.8	6.8
Leucine	g/kg	12.6	11.2	13.1	10.9	12.5	11.8	12.4	11.0
Histidine	g/kg	4.2	4.2	4.4	4.3	4.4	4.5	4.3	4.6
Glycine	g/kg	8.0	8.2	8.5	8.2	8.2	8.9	8.5	9.0
Serine	g/kg	8.1	6.8	8.5	6.7	8.3	7.6	8.2	7.2
Phenylalanine	g/kg	8.1	7.1	8.4	6.7	8.3	7.7	8.2	7.5
Alanine	g/kg	7.3	6.5	7.7	6.4	7.3	7.1	7.6	6.7
Proline	g/kg	9.1	11.3	9.7	10.8	11.6	10.3	11.2	9.9

SCP-SF = Standard Crude Protein and Standard Fiber (Control Diet); SCP-HF = Standard Crude Protein and High Fiber; LCP-SF= Low Crude Protein and Standard Fiber; LCP-HF = Low Crude Protein and High Fiber.

### Production performance and mortality

The production data included feed intake, egg production, and mortality. The number of eggs was recorded daily per pen. Egg weight was measured once per week, which involved all eggs from each pen being weighed. Production performance variables were calculated over 28-day periods. Feed intake was determined as the difference between the total feed offered and the residual feed remaining in the feeders, and average daily feed intake per hen was calculated based on the number of hens present in each pen, corrected for mortality. Mortality was recorded daily. Hen-day egg production was calculated as the total number of eggs produced divided by the cumulative number of hen-days, multiplied by 100, and egg mass was calculated by multiplying hen-day egg production by the average egg weight. Feed conversion ratio (FCR) was calculated by dividing average daily feed intake by egg mass. A daily routine inspection was conducted, and any hen seen to possess severe pecking injuries or an overall poor health condition was excluded from the trial. Excluded hens were categorized into two groups: euthanized and removed. Euthanized hens were those with critical injuries and an overall poor general condition that required immediate euthanasia. Removed hens refers to those that were transferred to a separate pen due to injuries and the risk of further deterioration.

### Egg quality

Egg quality was assessed at 35 and 48 weeks of age. For egg quality assessment on each time point, 10 eggs were randomly collected from the egg cradle outside the nest of each pen one day before the start of the testing period and stored at 4 °C until analysis. On both occasions, the tests were conducted over three days, with an equal number of eggs from each pen tested daily. Prior to testing each day, the eggs were brought to room temperature for one hour. Egg weight was measured using a Mettler PE 160 laboratory scale with an accuracy of 0.01 g. Eggshell breaking strength (kgF) was determined using an Egg Force Reader (Orka Food Technology Ltd., West Bountiful, UT). The egg content was poured onto a flat surface, and the albumen height (mm) was measured 0.5 cm from the yolk using an Ames S6428 micrometer (USA). The yolk was then separated from the albumen, and both components were weighed individually. Yolk color was evaluated using the Roche Yolk Color Fan (Switzerland). Eggshell weight was recorded after the shell membrane was removed. Shell thickness was measured at three equatorial points using a Mitutoyo Absolute digital micrometer (NO. 7360) after the membrane was removed. Albumen dry matter content was determined by drying the albumen at 60 °C overnight, followed by 24 h at 103 °C. After 36 h of drying, the samples were placed in a vacuum desiccator, and their weight was recorded after reaching room temperature.

### Body weight and integument condition scoring

Body weight measurement and integument condition scoring were performed twice, at 34 and 49 weeks of age. Twenty hens were randomly selected from each pen. Each hen was weighed before integument scoring. The scoring method followed the approach described by Tauson et al. (2005) and was based on a 1 to 4 scale, where 4 represented the best integument condition, 3 indicated good condition, scores ≤2 indicated severe damage, and a score of 1 represented very poor integument condition. Scoring of feather condition was performed on six body regions, including neck, back, tail, wings, breast, and cloaca. Furthermore, pecking injuries were evaluated on the comb, back, and feet using the same 1 to 4 scale as the feather scoring.

### Novel object test

The novel object test was performed on three occasions: at 37, 47, and 50 weeks of age by the same observer. Testing occurred in the afternoon between 1300 and 1700 h in the litter area of each pen. Before starting, the observer walked in front of all experimental pens for 10 min to acclimate the hens to their presence. The observer then slowly entered each pen and stood next to the observation area for three minutes to allow the hens to become comfortable. The novel object, a multicolored stick 20 cm in length and 2 cm in diameter, was then placed in the center of the observation area. Observations began immediately after the observer moved away from the object and stood in a corner outside the observation area. The number of hens within a one-hen length radius (∼35 cm) of the novel object was recorded every 10 s for two minutes.

### Pecking behavior

Pecking observations were performed on three occasions at 43, 46, and 47 weeks of age. All observations were conducted by a single observer. On each observation day, pecking behavior was recorded in all pens once in the morning (0800–1200 h) and once in the afternoon (1300–1700 h). Observations were made on the slatted area, excluding areas beneath the perches. Before each observation session, the observer walked along the aisle between the pens for 10 min to acclimate the hens to their presence. Observations were made in each pen in a randomized order. Additionally, before recording behavior in each pen, the observer stood outside the pen for 30 s to allow the hens to become comfortable with the presence of the observer. Each pen was continuously observed for 10 min. The hens were free to move between areas; therefore the number of hens present in the observation area was recorded every minute. The frequency of five types of pecking was recorded, and detailed descriptions of each type are provided in the ethogram (Table 5). Both single pecking and pecking bouts were observed. A peck was defined as a single directed peck toward another hen or a pecking object, and pecks separated by ≥10 s were considered independent events. Bouts were defined as continuous pecking at a single spot, regardless of intensity. If a continuous pecking episode was interrupted by a gap of 10 s or more, it was considered as one bout.

**Table 5:** Ethogram of pecking behaviors recorded in laying hens. The table summarizes both social interactions and pecking directed at environmental enrichments.

**Behavior**	**Description**
Aggressive Pecking	The bird directs one or more pecks toward the head region (from the comb to the base of the neck) of another bird, using a stabbing or pulling motion that provokes an immediate response from the recipient, such as moving away or loud vocalization.
Severe Pecking	The bird performs one or more pecks directed at the feathers or skin of another individual, targeting any body area except the head region (from the comb to the base of the neck), using a stabbing or pulling motion that elicits an immediate response from the recipient, such as withdrawal or vocalization.
Gentle Pecking	Pecking of a conspecific that does not cause damage or a reaction of the receiver.
Pecking on Peck Stone	Bird delivers one or more pecks directed at the destructible Peck Stone enrichment block composed of salts and minerals, provided in red-colored bowls.
Pecking on Concrete Block	Bird delivers one or more pecks directed at the non-destructible rectangular concrete block provided as an environmental enrichment.

### Statistical analysis

The production data were compiled into 28-day periods, commencing at 20 weeks of age and resulting in a total of eight periods. During weeks 40 to 43 of age, feed residues were measured after 30 days due to a deviation in measurement timing; therefore, the subsequent period, 44 to 47 weeks of age, consisted of 26 days. In addition, the final period (48–50 weeks of age) comprised 21 days due to the termination of the experiment at 50 weeks of age. Integument condition data were analyzed by aggregating all six feather scores (neck, back, tail, cloaca, wings, breast) into a composite score, whereas the three injury scores (comb, rear body, and foot) were analyzed separately. Mortality data were first summarized for the whole trial for each pen before statistical analysis was carried out. Data were analyzed using R (R Core Team, 2024, Version 4.4.2). All traits, except mortality data, were analyzed using linear mixed-effects models with dietary fiber, protein level, and age as fixed effects, including all possible interactions, and pen as a random effect. To account for the time series structure of the data, a temporal correlation coefficient was added to the error term; additionally, heterogeneity of variation for each period was allowed by estimating separate variances. For egg quality, measured at two discrete time points, a general symmetric correlation structure (corSymm) was applied to account for repeated measures within pens. For production performance, recorded over eight consecutive periods, a continuous autoregressive correlation structure (corCAR1) was used to model the temporal correlation of repeated measurements within pens. Model residuals were assessed for normality using Q-Q plots and histograms. Analysis of variance (ANOVA) was conducted on the fitted mixed models to test the significance of the fixed effects and their interactions. Post-hoc comparisons between groups were performed using the Tukey-Kramer test on estimated marginal means with the emmeans package (Lenth, 2023), and the differences were visualized using the compact letter display (CLD) function from the multcomp package. Mortality data were not normally distributed and were therefore analyzed using the non-parametric Kruskal–Wallis test. Due to the large number of zero observations which resulted in a non-normal data distribution, no statistical analysis was performed for pecking behavior. Instead, descriptive statistics were used, and the results are presented as peck rate per hen per hour. All other results are presented as mean ± standard error of the mean (SEM), and for mortality, both the mean and median are reported. Statistical significance was considered at p < 0.05.

## Results

### Mortality

Mortality was not significantly affected by fiber level, protein level, or treatment group (Table 6). By the end of the trial, 15.33% of the hens were no longer part of the study as they had been removed (5.17%), euthanized (3.76%), or found dead (6.40%). The main reasons for removal were pecking injuries, particularly on the feet, comb, head, and cloaca, as well as general weakness, small body size, or inability to compete within the group. Euthanasia was primarily performed in cases of severe injuries (e.g., extensive foot or head wounds, or cloacal pecking) where recovery was unlikely, and welfare was severely compromised.

**Table 6:** Mortality and Exclusion of Laying Hens Fed Diets Differing in Fiber and Crude Protein Levels from 20 to 50 Weeks of Age.

Factor	Level^1^	Dead^2^ (%)		Euthanized^3^ (%)		Removed^4^ (%)		Total Excluded^5^ (%)	
		Mean	Median	Mean	Median	Mean	Median	Mean	Median
Fiber	SF	7.76	4.43	3.20	3.45	5.29	5.90	16.24	15.28
	HF	4.43	3.45	3.32	2.96	4.80	4.45	12.55	10.83
Protein	SCP	6.89	4.90	3.32	2.48	5.90	7.35	16.12	13.79
	LCP	5.28	2.96	3.20	2.97	4.19	2.94	12.67	9.36
P-value									
Fiber		0.527		0.958		0.750		0.752	
Protein		0.051		0.792		0.184		0.206	
Treatment^6^		0.237		0.925		0.304		0.554	

1 SF = Standard Fiber; HF = High Fiber; SCP = Standard Crude Protein; LCP = Low Crude Protein.

2 Dead refers to hens found dead in the pens.

3 Euthanized refers to hens with severe injuries that necessitated humane euthanasia.

4 Removed refers to injured hens removed from the pen to prevent further deterioration of welfare.

5 Total Excluded represents the sum of dead, euthanized, and removed hens.

6 Treatment includes combinations of fiber and protein levels as follows: SCP-SF = Standard Crude Protein and Standard Fiber (Control Diet); SCP-HF = Standard Crude Protein and High Fiber; LCP-SF= Low Crude Protein and Standard Fiber; LCP-HF = Low Crude Protein and High Fiber.

Of the 104 hens that were found dead, cloacal cannibalism accounted for 48%, foot pecking for 31%, and unknown cause of death for 18%. Of the 61 euthanized hens, injuries caused by foot pecking accounted for 49%, followed by cloacal injuries (25%), and head injuries (19%). Of the 84 hens removed from the pens due to injuries, foot injuries were the most common reason (48%), followed by head injuries (29%), and injuries on the dorsal tail base (8%).

### Body weight and integument condition scoring

A significant fiber × age interaction was observed for body weight: at 34 weeks of age, body weight did not differ between hens fed HF and SF diets, whereas at 49 weeks of age, hens fed HF diets had higher body weight than those fed SF diets (Table 7). No main effects of fiber or protein level were detected. Age had a significant effect, with body weight increasing from 34 to 49 weeks.

**Table 7:** Body weight and integument condition of laying hens at 34 and 49 weeks of age in response to interaction effects of fiber × age and crude protein × age. For feather condition, scores from 6 body regions were summed, resulting in a total score ranging from 6 (worst) to 24 (best). Pecking injuries were scored on a scale from 1 (worst) to 4 (best).

**Factor**	**Level**^1^	**Age (wk)**	**Body Weight (g)**	**Feather Condition**^2^	**Pecking Injuries**^3^		
					Comb	Rear Body	Foot
Fiber	HF	34	1753 ± 12.9	22.8 ± 0.5	2.63 ± 0.06	3.98 ± 0.18^a^	3.18 ± 0.13
	SF	34	1755 ± 12.9	21.4 ± 0.5	2.46 ± 0.06	3.12 ± 0.18^b^	3.34 ± 0.13
	HF	49	1864 ± 14.6^a^	18.8 ± 0.5^a^	2.55 ± 0.06	3.29 ± 0.19	3.39 ± 0.13
	SF	49	1800 ± 14.6^b^	15.5 ± 0.5^b^	2.45 ± 0.06	2.94 ± 0.19	3.41 ± 0.13
Protein	LCP	34	1744 ± 12.9	22.0 ± 0.5	2.62 ± 0.06	3.58 ± 0.18	3.31 ± 0.13
	SCP	34	1764 ± 12.9	22.2 ± 0.5	2.46 ± 0.06	3.52 ± 0.18	3.21 ± 0.13
	LCP	49	1844 ± 14.6	16.8 ± 0.5	2.48 ± 0.06	3.01 ± 0.19	3.49 ± 0.13
	SCP	49	1821 ± 14.6	17.5 ± 0.5	2.52 ± 0.06	3.21 ± 0.19	3.30 ± 0.13
**P-value**							
Fiber			0.143	0.016	0.093	0.012	0.605
Protein			0.893	0.621	0.440	0.990	0.397
Age			<0.001	<0.001	0.396	<0.001	0.020
Fiber × Protein			0.524	0.774	0.794	0.947	0.904
Fiber × Age			0.003	<0.001	0.466	<0.001	0.213
Protein × Age			0.055	0.034^4^	0.052	0.029^5^	0.417
Fiber × Protein × Age			0.749	0.686	0.396	0.127	0.256

1 SF = Standard Fiber; HF = High Fiber; SCP = Standard Crude Protein; LCP = Low Crude Protein.

2 For feather condition, scores from 6 body regions were summed, resulting in a total score ranging from 6 (worst) to 24 (best).

3 Pecking injuries were scored on a scale from 1 (worst) to 4 (best).

4 Feather scores did not differ within individual weeks for the Protein × Age interaction after Tukey–Kramer adjustment.

5 Rear body injury scores did not differ within individual weeks for the Protein × Age interaction after Tukey–Kramer adjustment.

a,b Means within the same age with differing superscripts differ significantly.

A significant fiber × age interaction was observed for feather condition score. At 34 weeks of age, feather scores did not differ between hens fed HF and SF diets, whereas at 49 weeks, hens fed HF diets had better feather condition scores than those fed SF diets (Table 7). Although a main effect of fiber was detected, it was primarily driven by differences observed at 49 weeks. The protein × age interaction was also significant; however, post hoc comparisons using compact letter display (CLD) did not reveal significant pairwise differences.

A significant fiber × age interaction was observed for rear body injury score. At 34 weeks of age, hens fed HF diets had better rear body injury scores than those fed SF diets, whereas no difference was detected at 49 weeks (Table 7). Although a main effect of fiber was identified, this effect was primarily driven by differences at 34 weeks. Age also affected integument condition, as feather scores declined from 34 to 49 weeks of age (22.1 ± 0.3 vs. 17.2 ± 0.4), and rear body injury scores became poorer with age (3.55 ± 0.1 vs. 3.11 ± 0.1).

### Pecking behavior

**Fig. 2:**
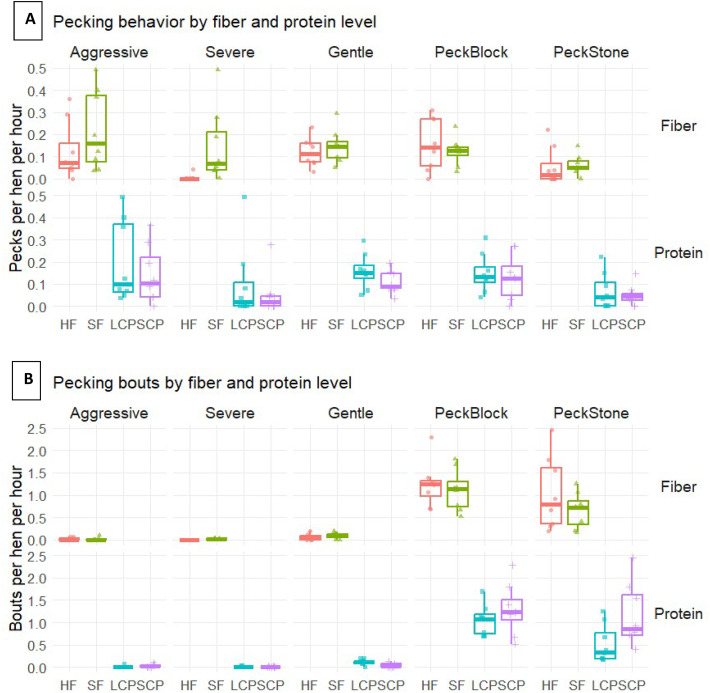
Distribution of pen-level mean pecking rates per hen per hour, recorded on three occasions at 43, 46, and 47 weeks of age, in laying hens fed diets differing in protein and fiber levels in a 2 × 2 factorial design. Both pecking (**A**) and pecking bouts (**B**) are shown. The boxes represent the interquartile range (IQR), the centre line indicates the median, whiskers extend to 1.5 × IQR, and points represent individual pens. **SF** = Standard Fiber; **HF** = High Fiber; **SCP** = Standard Crude Protein; **LCP** = Low Crude Protein.

The distribution of pen-level pecking and bout rates is illustrated in Fig. 2. Given that the data were highly zero-inflated, and the median was zero for several behaviors, the results are presented descriptively as mean pecking or bout rates per hen per hour with the associated standard deviation (SD). Detailed numerical summaries are provided in the Supplementary Tables (Tables 3 and 4).

Overall, pecking events observed in the morning averaged 0.68 pecks per hen per hour (SD = 0.39), whereas in the afternoon, they averaged 0.48 pecks per hen per hour (SD = 0.27). The number of bouts directed at the Peck Block and Peck Stone was numerically higher than those directed at conspecifics (Fig. 2B). Bouts were more often observed in the afternoon (3.45 bouts per hen per hour, SD = 2.07) compared with the morning (0.78 bouts per hen per hour, SD = 0.48).

Severe pecking was numerically lower among hens under the HF diet, with 0.01 pecks per hen per hour (SD = 0.02), compared with 0.15 pecks per hen per hour (SD = 0.17) in the SF diet. For aggressive pecking, HF showed 0.12 pecks per hen per hour (SD = 0.13), whereas SF showed 0.22 pecks per hen per hour (SD = 0.18). Regarding dietary CP levels, the aggressive pecking rate was 0.20 pecks per hen per hour (SD = 0.18) for the LCP diets and 0.14 pecks per hen per hour (SD = 0.13) for the SCP diets. Similarly, for severe pecking, the LCP group had 0.10 pecks per hen per hour (SD = 0.17), whereas the SCP group had 0.05 pecks per hen per hour (SD = 0.09).

### Novel object test

There was no effect of fiber or protein level on the proportion of hens approaching the novel object, and no significant interaction was observed (P > 0.05). However, a significant effect of age was found, with the proportion of hens approaching the novel object decreasing as the hens aged. Mean proportion values for each dietary factor and inclusion level are provided in Table 8.

**Table 8:** Novel Object Test results presented as mean proportion of hens approaching novel object at each age in response to different dietary levels of crude protein and fiber.

**Factor**	**Level**^1^	**Week 37**	**Week 47**	**Week 50**
Fiber	HF	0.137 ± 0.018	0.089 ± 0.012	0.070 ± 0.015
	SF	0.126 ± 0.018	0.087 ± 0.012	0.071 ± 0.015
Protein	LCP	0.136 ± 0.018	0.089 ± 0.012	0.070 ± 0.015
	SCP	0.127 ± 0.018	0.087 ± 0.012	0.072 ± 0.015
P-value				
Fiber			0.881	
Protein			0.889	
Age			0.001	
Fiber × Protein			0.799	
Fiber × Age			0.892	
Protein × Age			0.913	
Fiber × Protein × Age			0.264	

1 SF = Standard Fiber; HF = High Fiber; SCP = Standard Crude Protein; LCP = Low Crude Protein.

### Production performance

Significant fiber × protein × period interactions were observed for feed intake and FCR (Table 9). Feed intake was generally unaffected by dietary treatments across most periods; however, during weeks 40-43, hens fed the SCP-SF diet consumed more feed than those fed SCP-HF and LCP-HF diets (130.5 vs. 121.2 and 120.8 ± 1.96 g, respectively). Similarly, FCR differed among treatments primarily during weeks 40-43, with hens fed SCP-HF (1.93) and LCP-HF (1.92) showing lower FCR than those fed SCP-SF (2.10) and LCP-SF (2.08; pooled SEM = 0.032). In the final period (weeks 47–50), hens fed LCP–SF had higher FCR (2.02) than those fed SCP-HF (1.82) and LCP–HF (1.78; pooled SEM = 0.048).

**Table 9:** Production performance of laying hens in response to fiber and crude protein levels in the diets across eight consecutive periods from 20 to 50 weeks of age.

Factor	Level^1^	Feed Intake^2^ (g/hen/d)	Egg Weight	Hen Day Egg Production^3^	Egg Mass	FCR^4^
			**(g)**	**(%)**	**(g/hen/d)**	
Fiber	SF	118	61.26^b^	92.5	57.13^b^	2.14
	HF	114	61.67^a^	92.9	57.74^a^	2.05
	SEM	1.4	0.10	0.2	0.13	0.02
Protein	SCP	116	61.66^a^	92.7	57.57	2.08
	LCP	116	61.28^b^	92.8	57.29	2.10
	SEM	1.4	0.10	0.2	0.13	0.02
P-Value						
Fiber		0.989	0.010	0.204	0.011	0.076
Protein		0.148	0.015	0.807	0.109	0.773
Period^5^		<0.001	<0.001	<0.001	<0.001	<0.001
Fiber × Protein^6^		0.123	0.582	0.451	0.228	0.166
Fiber × Period^6^		<0.001	0.069	0.773	0.409	<0.001
Protein × Period^6^		0.216	0.226	0.837	0.845	0.093
Fiber × Protein × Period^6^		0.001	0.678	0.928	0.920	0.018

1 SF = Standard Fiber; HF = High Fiber; SCP = Standard Crude Protein; LCP = Low Crude Protein.

2 Feed intake was calculated on a hen-day basis, accounting for mortality and removals within each pen during the experimental period.

3 Laying percentage was calculated on a hen-day basis using the number of hens present in each pen, accounting for mortality and removals.

4 Feed conversion ratio calculated using hen-day adjusted feed intake and egg mass values, accounting for mortality and removals.

5 Production performance parameters were measured across eight consecutive periods from 20 to 50 weeks of age (20–23, 24–27, 28–31, 32–35, 36–39, 40–43, 44–47, and 48–50 weeks of age).

6 Significant Fiber × Period, Protein × Period, and Fiber × Protein × Period interactions are described in the Results section, and detailed interaction tables are provided in the supplementary material (Supplementary Tables 7 and 8).

a,b Different superscripts within a column indicate significant differences between dietary crude protein or fiber levels.

A fiber × period interaction was also detected for feed intake and FCR. Feed intake was lower among hens fed HF diets than SF diets during weeks 32–35, 36–39, and 40–43 (116.1 vs. 121.8 ± 1.53 g; 114.2 vs. 119.2 ± 1.45 g; 121.0 vs. 129.7 ± 1.39 g, respectively). Regarding FCR, no differences were observed during the first three periods, whereas in the subsequent periods HF diets consistently resulted in lower FCR compared with SF diets.

Egg weight was affected by both fiber and protein level, with hens fed HF diets producing heavier eggs than those fed SF diets and hens fed SCP diets producing heavier eggs than those fed LCP diets (Table 9). Egg mass was also greater among hens fed HF diets than in those fed SF diets. No treatment effects were observed for hen-day egg production. Period significantly influenced all production performance traits (P < 0.001).

### Egg quality

A significant fiber × protein × age interaction was detected for albumen height; however, Tukey–Kramer post hoc comparisons did not reveal significant differences among dietary treatments within individual ages. Yolk color was affected by a protein × age interaction, with no differences between protein levels at 35 weeks, whereas at 48 weeks, hens fed LCP diets had higher yolk color scores than those fed SCP diets (Table 10). A significant Fiber × Protein interaction was observed for yolk color, where hens fed the LCP-SF diet had significantly higher yolk color than hens fed the SCP-SF, SCP-HF, and LCP-HF diets (12.74 vs. 11.79, 11.90, and 12.00 ± 0.083, respectively). In addition, main effects were observed for certain parameters. Shell breaking strength was greater among hens fed LCP diets than in those fed SCP diets (4.89 vs. 4.64 ± 0.06 kg). Yolk color was lower among hens fed HF diets compared with SF diets (11.9 vs. 12.3 ± 0.1), and higher for hens fed LCP diets than SCP diets (12.4 vs. 11.8 ± 0.1). No main effects of insoluble fiber or protein were detected for egg weight, albumen dry matter, yolk weight, shell thickness, shell weight, or Haugh unit.

**Table 10:** Egg quality parameters of laying hens at 35 and 48 weeks of age in response to interaction effects of fiber × age and crude protein × age.

**Factor**	**Level**^1^	**Age (wk)**	**Egg Weight (g)**	**Breaking Strength (KgF)**	**Shell Thickness (mm)**	**Shell Weight (g)**	**Yolk Color**	**Yolk Weight (g)**	**Albumen Height (mm)**	**Albumen Weight (g)**	**Albumen DM (%)**	**Haugh Unit**
Fiber	HF	35	64.6 ± 0.4	4.95 ± 0.08	0.389 ± 0.002	6.77 ± 0.06	12.1 ± 0.1	16.0 ± 0.1	9.79 ± 0.07	39.4 ± 0.3	11.6 ± 0.1	97.2 ± 0.4
	SF	35	63.9 ± 0.4	4.85 ± 0.08	0.391 ± 0.002	6.78 ± 0.06	12.3 ± 0.1	16.1 ± 0.1	9.62 ± 0.07	38.7 ± ± 0.3	11.7 ± 0.1	96.5 ± 0.4
	HF	48	66.0 ± 0.6	4.62 ± 0.05	0.370 ± 0.002	6.43 ± 0.05	11.8 ± 0.1	17.3 ± 0.2	9.35 ± 0.08	39.3 ± 0.5	11.1 ± 0.1	94.9 ± 0.4
	SF	48	65.4 ± 0.6	4.63 ± 0.05	0.369 ± 0.002	6.37 ± 0.05	12.2 ± 0.1	17.4 ± 0.2	9.14 ± 0.08	38.8 ± 0.5	11.2 ± 0.1	93.9 ± 0.4
Protein	LCP	35	64.2 ± 0.4	5.00 ± 0.08	0.392 ± 0.002	6.78 ± 0.06	12.4 ± 0.1	16.1 ± 0.1	9.72 ± 0.07	38.8 ± 0.3	11.7 ± 0.1	97.0 ± 0.4
	SCP	35	64.4 ± 0.4	4.80 ± 0.08	0.389 ± 0.002	6.77 ± 0.06	12.1 ± 0.1	15.9 ± 0.1	9.69 ± 0.07	39.3 ± 0.3	11.6 ± 0.1	96.7 ± 0.4
	LCP	48	65.8 ± 0.6	4.77 ± 0.05	0.370 ± 0.002	6.42 ± 0.05	12.3 ± 0.1^a^	17.5 ± 0.2	9.18 ± 0.08	39.0 ± 0.5	11.2 ± 0.1	94.0 ± 0.4
	SCP	48	65.5 ± 0.6	4.48 ± 0.05	0.369 ± 0.002	6.37 ± 0.05	11.6 ± 0.1^b^	17.2 ± 0.2	9.31 ± 0.08	39.1 ± 0.5	11.2 ± 0.1	94.7 ± 0.4
P-value												
Fiber			0.136	0.928	0.855	0.577	0.004	0.699	0.062	0.038	0.159	0.126
Protein			0.866	0.001	0.382	0.505	<0.001	0.114	0.698	0.218	0.457	0.768
Age			0.014	<0.001	<0.001	<0.001	0.003	<0.001	<0.001	0.992	<0.001	<0.001
Fiber × Protein^2^			0.742	0.191	0.988	0.661	<0.001	0.596	0.329	0.934	0.484	0.300
Fiber × Age			0.888	0.303	0.504	0.500	0.135	0.872	0.691	0.845	0.956	0.617
Protein × Age^2^			0.630	0.407	0.507	0.735	0.008	0.721	0.212	0.701	0.738	0.119
Fiber × Protein × Age^2^			0.169	0.985	0.346	0.884	1.000	0.545	0.011	0.152	0.603	0.015

1 SF = Standard Fiber; HF = High Fiber; SCP = Standard Crude Protein; LCP = Low Crude Protein.

2 Significant interactions are described in the Results section, and detailed interaction data are provided in the Supplementary Material (Supplementary Table 5).

a,b Means within a week with differing superscripts differ significantly.

All egg quality parameters, except albumen weight, were significantly affected by age. From 35 to 48 weeks of age, egg weight and yolk weight increased (64.27 vs. 65.67 g and 16.01 vs. 17.34 g, respectively), whereas breaking strength, shell thickness, shell weight, albumen height, yolk color, and Haugh unit decreased (4.90 vs. 4.62, 0.390 vs. 0.369 mm, 6.77 vs. 6.40 g, 9.70 vs. 9.24, 12.23 vs. 11.96, and 96.83 vs. 94.34, respectively).

## Discussion

The present study demonstrated that dietary crude protein and fiber levels influenced feather condition, pecking injuries, selected production parameters, and egg quality traits. A relatively high number of laying hens were excluded from the experiment due to mortality and culling, which could potentially raise questions regarding the practical applicability of the experimental diets and the potential bias introduced in production performance and integument condition assessments. However, there was no difference in the total number of excluded hens between hens fed the adjusted or standard levels of protein and fiber, indicating it is unlikely that the problem was linked to dietary treatment. Therefore, any potential bias caused by the exclusion of hens was distributed among all treatments. Early mortalities and removals were primarily due to head and toe pecking. Toe pecking is a multifactorial problem that may be initiated by barn equipment (e.g., metal slats) or stress during transfer to the laying facility, and it is predominantly observed among white laying hens (Gebhardt-Henrich et al., 2023). The incidence of toe pecking appears to be increasing in countries where beak trimming is not practiced, and diet particle size has been reported to influence this behavior, with higher toe pecking observed among hens fed coarse mash compared with fine mash diets (Mens et al., 2023). This may be related to altered feeding behavior, as birds consuming coarse diets may spend less time feeding and therefore more frequently engage in redirected pecking activity. Aggressive head pecking, on the other hand, is associated with the establishment of social hierarchy (Rodenburg et al., 2013). Group size is a factor that is recognized to influence the occurrence of aggressive behavior or feather pecking among laying hens. Bilčík & Keeling (2000) reported that aggressive pecking increased with increasing group size when hens were kept in groups of 15, 30, 60, and 120 on deep litter at the same stocking density. The authors discussed that while such pecking behaviors are not overly common in large commercial flocks, group size still has an effect. One possible explanation is that birds kept in large commercial flocks may adopt different strategies to establish social relationships (Pagel and Dawkins, 1997). In the present study, the hens were housed in groups of 100, which may have contributed to the pecking-related head injuries. Cloacal pecking was also identified as a cause of mortality. Notably, Allen & Perry (1975) reported an effect of group size on cloacal pecking. Although their study was conducted in cages with much smaller groups of 3, 4, or 6 hens, mortality due to cannibalism increased with increasing group size. Furthermore, feather pecking has also been shown to potentially lead to cloacal pecking (Lambton et al., 2015).

In contrast to mortality, the present study showed that feather condition was affected by fiber level and laying hens fed high-fiber diets had improved feather condition compared with hens fed standard-fiber diets. The inclusion of oat hulls increased the level of insoluble fiber in the high-fiber diets. The beneficial role of oat hulls in mitigating feather pecking and improving plumage has been acknowledged since early feeding experiments (Bearse et al., 1940). More recent studies have confirmed these beneficial effects of higher levels of insoluble fiber (Steenfeldt et al., 2007; Kalmendal and Wall, 2012; Kalmendal and Bessei, 2012; Qaisrani et al., 2013; Patt et al., 2022). Several mechanisms may explain why higher inclusion of insoluble fiber improves feather condition of laying hens. Insoluble NSP may prolong the retention time in the foregut, resulting in increased satiety (van Krimpen et al., 2007), which may in turn reduce pecking behavior. Feather consumption itself has been shown to produce a similar satiety effect (Harlander-Matauschek et al., 2006), supporting the idea that enhanced satiety reduces pecking behavior. However, the effect of insoluble NSP on gizzard and gut function is partly linked to particle size, where coarser particles are retained and stimulate the gizzard to a greater extent (Hetland et al., 2004; van Krimpen et al., 2009). In the present study, the oat hulls were grinded and incorporated into the pelleted diets. Therefore, it is unlikely that the observed effect of insoluble NSP was due to physical stimulation of the gizzard. One established theory is that feather pecking is a redirected foraging behavior (Blokhuis, 1986). Red junglefowl, the ancestor of domestic chickens, spend approximately 50% of their active time foraging for food (Savory, 2010). In the study by Steenfeldt et al. (2007), fiber-rich foraging materials such as silages and carrots reduced severe feather pecking and improved plumage, likely by stimulating foraging activity. However, in the present study, oat hulls were directly incorporated into pelleted diets. Higher levels of insoluble NSP are known to increase feeding time by lowering the eating rate (van Krimpen et al., 2007). Consequently, hens consume the same amount of feed at a slower rate, meaning that they spend a longer time at the feeder, which may better satisfy their motivation to perform feed-related behaviors and reduce redirected pecking at conspecifics. In the current study, although no overall main effect of fiber level was observed for feed intake, the significant Fiber × Period interaction showed that feed intake was lower in the HF group than in the SF group during three consecutive periods (32–43 weeks of age). Despite the lower intake during those periods, the HF group demonstrated better feather scores. The potentially extended feeding time and greater satisfaction of feeding behavior associated with high-fiber diets may provide a plausible explanation for the improved feather condition observed in the HF group. Although high fiber resulted in better feather condition scores, feather and toe pecking were observed across all treatments, as reflected in the high mortality in all treatments. Housing during rearing has been reported to have a significant impact on feather condition later in life (Johnsen et al., 1998). In the present study, pullets were reared in a multi-tier aviary system and subsequently housed in a single-tier system during the trial. This transition may have contributed to the occurrence of feather pecking across all groups. Moreover, red mites were observed in the barn, and infestations are known to be associated with reduced weight gain and egg production, anemia, scratching, feather pecking, cannibalism, and mortality (Kilpinen et al., 2005; Sigognault Flochlay et al., 2017). However, since the mites were controlled through timely interventions it is unlikely that they were a contributing factor to the feather pecking and pecking-related injuries recorded in this study.

Provision of higher levels of insoluble NSP lowered the incidence of rear body injuries around the cloaca. Hartini et al. (2002) reported a significant reduction in cannibalism in ISA Brown layers with the provision of insoluble fiber during the pre-lay and early laying periods, as reflected in the fewer mortalities attributed to cannibalism. It has been reported that damaged feathers can trigger the initiation of cannibalism (McAdie and Keeling, 2000). In the present study, hens with well-feathered back regions did not exhibit pecking injuries; however, as feather pecking escalates and the area becomes partially or fully denuded, pecking is redirected towards the skin, leading to injuries (Rodenburg et al., 2013).

In our study, interpretation of pecking behavior is limited because the high number of zero observations precluded statistical analysis. Therefore, it remains unclear whether the observed numerical differences reflect true treatment effects, despite patterns consistent with integument condition scores. The relatively low overall peck rate observed in the present study may be explained by the few sampling occasions and short recording duration during each observation period. A greater proportion of pecking bouts were observed on the pecking stone in the afternoon compared with the morning, similar to the findings of McAuley et al. (2025). These pecking stones were mineral blocks containing calcium, which may indicate a physiological need for increased calcium to support eggshell formation (Hughes, 1972; Mongin and Sauveur, 1974). Between the two pecking materials, the hens exhibited a higher pecking rate toward the concrete block compared with the destructible pecking stone, which contrasts with the findings of McAuley et al. (2025), who reported laying hens to have a higher preference for softer mineral-based pecking stones. This difference may be related to the larger size of the concrete block compared with the pecking stone.

Lowering crude protein or increasing insoluble fiber in the diet did not affect the proportion of hens approaching the novel object. Although higher inclusion of insoluble fiber improved feather condition and reduced rear body injuries, it did not affect fearfulness, contrary to our hypothesis. Unlike studies where fiber was provided as enrichment and enhanced foraging behavior (Aerni et al., 2000), oat hulls incorporated into a pelleted diet may not sufficiently stimulate exploration behavior to reduce fear responses (Jones, 1982; Ben-Mabrouk et al., 2022). Providing fiber separately rather than incorporated into pelleted feed may be more effective in stimulating foraging behavior and serve as environmental enrichment, thereby potentially reducing fearfulness (El-Lethey et al., 2000). In addition, dietary fiber may indirectly influence behavior by modulating gut health and microbiota composition; however, this mechanism was not evaluated in the present study. Cecal samples collected from the same hens will be analyzed in a separate study to further investigate the potential effects of fiber on the cecal microbiota. Notably, the proportion of hens approaching the novel object progressively decreased from 34 to 50 weeks of age, suggesting that fearfulness increased with age. Previous studies have reported that increased fearfulness in birds is associated with higher incidences of feather pecking and poorer plumage condition (Jones, 1996; Rodenburg et al., 2004; Uitdehaag et al., 2008). In the current study, feather condition deteriorated with age, and the frequency of rear body pecking injuries and mortality related to pecking behavior remained high until the end of the experiment. These findings suggest that increased fearfulness may have contributed to the reduced proportion of hens approaching the novel object as they aged. The results are in agreement with Hüttner et al. (2023), who also demonstrated that increased feather damage and cannibalism are associated with elevated fear responses. As the fear-inducing nature of an event is largely determined by its novelty (Forkman et al., 2007), it could be assumed that repeated testing reduced the object’s novelty and hens became habituated over time. However, this explanation appears less likely given that previous studies found the opposite trend. Jones (1988) reported that avoidance of novel objects decreased across three consecutive days at 26 weeks of age, and Hocking et al. (2001) observed reduced avoidance with age from 0 to 31 weeks of life. Another possible explanation is that the hens in the previous studies were younger than those in the present study, and the exploration behavior of older hens may have declined with age. Thus, the decline in the proportion of hens approaching the novel object with advancing age likely reflects an age-related increase in fearfulness and reduced motivation for exploration, potentially reinforced by worsening feather condition and ongoing social stress within the flock.

Egg weight was significantly influenced by both protein and fiber levels: hens fed the LCP diets produced lighter eggs, while those on the high-fiber diets produced heavier eggs and consequently a higher egg mass. The effect of amino acids supplementation in LCP diets on egg weight has been reported inconsistently in the literature, with some studies noting a reduction in egg weight (Halle, 2002; Valkonen et al., 2006; Novak et al., 2006; Ji et al., 2014; Harn et al., 2021) and others finding no effect (Roberts et al., 2007a; Rama Rao et al., 2011; Burley et al., 2013). Our findings, together with earlier reports, indicate that egg weight is more sensitive to dietary CP reduction than laying rate. Leeson & Caston (1996) suggested that reduced egg weight in response to LCP diets can be attributed to insufficient total nitrogen, which can lead to lower albumen weight (Penz and Jensen, 1991). The different mechanisms of protein deposition in yolk and albumen may explain this sensitivity: yolk proteins are synthesized in the liver and continuously deposited in the ovum, whereas albumen proteins are deposited over a short period of ∼3 hours (Sah and Mishra, 2018). This generates an acute demand for circulating amino acids, and a limited nitrogen pool in LCP diets may restrict albumen synthesis, thereby reducing egg weight (Penz and Jensen, 1991). This pattern also suggests that supplementation with free amino acids may not fully support protein synthesis in the magnum, where albumen is deposited. Osonka et al. (1947) reported that peptide-bound methionine had a greater effect on egg protein deposition compared with the free form, likely because the peptide form provided a more effective amino acid supply during the critical post-ovulation window. Similarly, Ji et al. (2014) found that hens fed LCP diets balanced for digestible amino acids still produced lighter eggs than those on higher-CP diets, despite equivalent amino acid levels. Together, these findings support the idea that a lower nitrogen pool in LCP diets may result in insufficient non-essential amino acids. Thus, inclusion of crystalline non-essential amino acids may help to maintain egg weight under reduced-CP conditions. Some recent studies have suggested that free and protein-bound amino acids differ in their digestive dynamics in broiler chickens (Liu and Selle, 2017; Selle and Liu, 2019), but whether similar effects occur in laying hens remains to be investigated.

Hens fed the HF diets produced significantly heavier eggs compared with those on the SF diets, which also translated into a higher daily egg mass. This finding is in line with Sozcu & Ipek (2020), who showed that higher inclusion of insoluble fiber can increase egg weight among laying hens. Since egg weight is often associated with body weight (Desbruslais et al., 2021), the significantly higher body weight observed in the HF group at 49 weeks of age may explain the greater egg weight reported in the present study. Insoluble fiber has been shown to promote gizzard development (González-Alvarado et al., 2010), leading to improved mechanical breakdown of feed and more efficient digestion. Higher insoluble fiber has been shown to enhance intestinal mucosal development by increasing villus dimensions (Röhe et al., 2019; Sozcu and Ipek, 2020), which is associated with improved absorptive capacity (Iji et al., 2001). This may have contributed to enhanced nutrient absorption among HF-fed hens, potentially explaining the higher body weight at 49 weeks and increased egg weight observed in the present study. Although the diets were formulated to be isoenergetic, the HF diets required the inclusion of additional oil to balance energy. Previous studies have shown that supplemental dietary fat can increase egg weight (Hoyle and Garlich, 1987). However, Mannion et al. (1992) reported that the linoleic acid component of dietary oils was primarily responsible for the increased egg weight observed with supplemental fat. Similarly, Grobas et al. (1999) discussed that hens respond rapidly to linoleic acid supplementation when diets are deficient in linoleic acid, regardless of dietary fat content. In the present study, linoleic acid levels were kept similar across diets to minimize the confounding effect of additional oil on egg weight. Even though the additional oil may still have influenced egg weight in the high-fiber diets, the individual contributions of fiber and supplemental oil cannot be separated within the scope of this study because both factors varied simultaneously.

A fiber × protein × age interaction was observed for feed intake, with hens fed the SCP-SF diet consuming more feed than hens fed the high-fiber diets (SCP-HF and LCP-HF) during only one period (40–43 weeks of age). Overall, feed intake tended to be lower among hens fed high-fiber diets from 32 to 43 weeks of age; however, the reason for the higher feed intake observed in SCP-SF hens during the 40–43-week period remains unclear.

Most egg quality traits remained unaffected by both protein and fiber levels, except for breaking strength and yolk color. The darker yolk color of eggs from the hens fed LCP diets is likely due to the higher corn content, and thus greater xanthophyll concentrations, compared with SCP diets (Peterson et al., 1939). Previous LCP studies with higher inclusion of corn have reported similar findings (Karunajeewa, 1972; Gunawardana et al., 2008; Asghar Saki et al., 2012; Ji et al., 2014). In contrast, higher fiber significantly reduced yolk color in the present study compared to the SF diets. Previous studies have shown that insoluble fiber can alter digesta passage rate, and it has also been suggested that fiber may act as a physical barrier that influences nutrient availability (Hetland et al., 2004). Therefore, the lower yolk color observed in HF diets may have been related to reduced release and absorption of carotenoids. Additionally, the Fiber × Protein interaction showed that yolk color differed among fiber-protein combinations, with the LCP-SF diet resulting in higher yolk color than the SCP-SF, SCP-HF, and LCP-HF diets. The higher corn inclusion in the phase 2 diet of the LCP-SF treatment compared with the other diets may have contributed to the higher yolk color observed in hens fed the LCP-SF diet.

The increase in eggshell breaking strength observed among hens fed LCP diets in the present study contrasts with most previous reports. Earlier studies have generally shown either no effect of LCP diets on breaking strength (Kang et al., 1996; Mousavi et al., 2013; Ji et al., 2014; Parenteau et al., 2020) or even a reduction in shell strength when hens were fed LCP diets ranging from 14.7% to 12.0% CP (Harn et al., 2021). As reported in the production performance results, the LCP diets led to lower egg weight, which can influence breaking strength. Kocevski et al. (2011) reported that increased egg weight is associated with reduced eggshell strength. Casiraghi et al. (2005) also found a correlation between lower egg weight and higher breaking strength, and the authors reported that breaking strength is positively correlated with eggshell thickness. Eggshells primarily consist of calcium carbonate (≈96%), with the remainder made up of organic matrix, magnesium, phosphorus, and trace elements (Nys et al., 2004). Another possible explanation is that the LCP diet used in this study contained higher levels of synthetic amino acids, which may have enhanced nutrient utilization efficiency and, in turn, supported mineral metabolism and shell deposition. Supporting this idea, Gül et al. (2024) reported that dietary threonine supplementation in laying quails improved shell quality by increasing serum phosphorus levels. Stronger eggshells are economically important, as breakage during collection and transport results in substantial financial losses (Hamilton, 1982). Therefore, the improved eggshell breaking strength observed with LCP diets may have practical relevance, although the underlying mechanisms require further investigation.

## Conclusion

This study contributes to the limited body of research examining the combined effects of reduced crude protein and high fiber levels under non-cage housing conditions and larger experimental group sizes on the production performance, egg quality, integument condition, and behavior of laying hens. High-fiber diets improved feather condition scores and reduced rear-body pecking injuries, while low crude protein lowered egg weight and increased eggshell breaking strength without compromising lay percentage. Our findings suggest that standard fiber levels in commercial diets may be suboptimal for maintaining feather integrity, particularly during periods of increased stress. A 2% unit increase in insoluble fiber, when properly formulated, can enhance welfare without compromising production. However, the exact mechanisms through which insoluble fiber improves feather condition remain unclear, and further research is needed to elucidate these underlying processes.

## Conflict of interest

The authors have no conflicts of interest to declare.

## CRediT authorship contribution statement

**Muhammad Adnan Aslam:** Writing – review & editing, Writing – original draft, Visualization, Methodology, Investigation, Formal analysis, Data curation. **Helena Wall:** Writing – review & editing, Validation, Supervision, Resources, Methodology, Funding acquisition. **Anette Wichman:** Writing – review & editing, Validation, Supervision, Methodology. **Emma Ivarsson:** Writing – review & editing, Validation, Supervision, Resources, Project administration, Methodology, Investigation, Funding acquisition, Conceptualization.

## Appendix Supplementary materials

Image, application 1

## Footnotes

Supplementary material associated with this article can be found, in the online version, at doi:10.1016/j.psj.2026.107369.
