# Supplementary material for: Effects of low crude protein and high-fiber diets on production performance, egg quality, behavior, and integument condition in laying hens
Source: Poult Sci. 2026 Jul 1;105(10):107369. doi: 10.1016/j.psj.2026.107369 (PMC13392609; doi:10.1016/j.psj.2026.107369)
Supplement: Supplementary file 1 [file mmc1.docx]

**Supplementary Tables**

Table 1: Fiber × protein and fiber × protein × age interaction effects on integument condition measured at 34 and 49 weeks of age.

| Treatment^1^ | Feather^2^ Condition | Pecking Injuries^3^ | | |
| --- | --- | --- | --- | --- |
|  |  | **Comb** | **Rear Body** | **Foot** |
| Fiber × Protein | | | | |
| SCP-SF | 18.8 | 2.43 | 3.09 | 3.31 |
| SCP-HF | 20.9 | 2.55 | 3.65 | 3.20 |
| LCP-SF | 18.1 | 2.48 | 2.98 | 3.44 |
| LCP-HF | 20.7 | 2.63 | 3.61 | 3.37 |
| SEM | 0.7 | 0.08 | 0.26 | 0.17 |
| Fiber × Protein × Age | |  |  |  |
| Week 34 |  |  |  |  |
| SCP-SF  SCP-HF  LCP-SF  LCP-HF | 21.6  22.8  21.2  22.8 | 2.36  2.56  2.55  2.70 | 3.08  3.98  3.17  3.98 | 3.34  3.09  3.35  3.27 |
| SEM | 0.7 | 0.09 | 0.26 | 0.18 |
| Week 49 | | | | |
| SCP-SF  SCP-HF  LCP-SF  LCP-HF | 16.0  19.0  15.0  18.6 | 2.50  2.54  2.40  2.56 | 3.10  3.33  2.77  3.25 | 3.29  3.31  3.52  3.46 |
| SEM | 0.7 | 0.09 | 0.27 | 0.18 |
| P Value | | | | |
| Fiber (F) | 0.016 | 0.093 | 0.012 | 0.605 |
| Protein (P) | 0.621 | 0.440 | 0.990 | 0.397 |
| Age (A) | <0.001 | 0.396 | <0.001 | 0.020 |
| F x P | 0.774 | 0.794 | 0.947 | 0.904 |
| F x A | <0.001 | 0.466 | <0.001 | 0.213 |
| P x A | 0.034 | 0.052 | 0.029 | 0.417 |
| F x P x A | 0.686 | 0.396 | 0.127 | 0.256 |

^1^SCP-SF = Standard Crude Protein and Standard Fiber (Control Diet); SCP-HF = Standard Crude Protein and High Fiber; LCP-SF= Low Crude Protein and Standard Fiber; LCP-HF = Low Crude Protein and High Fiber

^2^For feather condition, scores from 6 body regions were summed, resulting in a total score ranging from 6 (worst) to 24 (best).

^3^Pecking injuries were scored on a scale from 1 (worst) to 4 (best).

Table 2: Fiber × protein and fiber × protein × age interaction effects on body weight measured at 34 and 49 weeks of age.

| Treatment^1^ | Body Weight (g) |
| --- | --- |
|  |  |
| Fiber × Protein |  |
| SCP-SF | 1771 |
| SCP-HF | 1813 |
| LCP-SF | 1784 |
| LCP-HF | 1804 |
| SEM | 16.0 |
| Fiber × Protein × Age |  |
| Week 34 |  |
| SCP-SF | 1761 |
| SCP-HF  LCP-SF  LCP-HF | 1767  1749  1740 |
| SEM | 18.2 |
| Week 49 |  |
| SCP-SF | 1781 |
| SCP-HF  LCP-SF  LCP-HF | 1860  1819  1868 |
| SEM | 20.6 |
| P Value |  |
| Fiber | 0.143 |
| Protein | 0.893 |
| Age (A) | <0.001 |
| F x P | 0.524 |
| F x A | 0.003 |
| P x A | 0.055 |
| F x P x A | 0.749 |

^1^SCP-SF = Standard Crude Protein and Standard Fiber (Control Diet); SCP-HF = Standard Crude Protein and High Fiber; LCP-SF= Low Crude Protein and Standard Fiber; LCP-HF = Low Crude Protein and High Fiber

Table 3: Mean pecking rate (pecks/hen/h) of laying hens fed diets with differing protein and fiber levels, observed at 43, 46, and 47 weeks of age. Values are presented as means ± standard deviation (SD).

| **Behavior** | **Type** | **Fiber**^1^ | | **Protein**^2^ | |
| --- | --- | --- | --- | --- | --- |
|  |  | SF | HF | SCP | LCP |
| Aggressive | Pecking | 0.22±0.18 | 0.12±0.13 | 0.14±0.13 | 0.20±0.18 |
| Severe | Pecking | 0.15±0.17 | 0.01±0.02 | 0.05±0.09 | 0.10±0.17 |
| Gentle | Pecking | 0.15±0.08 | 0.12±0.07 | 0.11±0.05 | 0.16±0.08 |
| Peck Block | Pecking | 0.12±0.06 | 0.15±0.12 | 0.13±0.10 | 0.15±0.09 |
| Peck Stone | Pecking | 0.06±0.04 | 0.06±0.08 | 0.05±0.05 | 0.07±0.08 |

^1^SF = Standard Fiber; HF = High Fiber

^2^SCP = Standard Crude Protein; LCP = Low Crude Protein

Table 4: Mean Bout rate (Bouts/hen/h) of laying hens fed diets with differing protein and fiber levels, observed at 43, 46, and 47 weeks of age. Values are presented as means ± standard deviation (SD).

| **Behavior** | **Type** | **Fiber**^1^ | | **Protein**^2^ | |
| --- | --- | --- | --- | --- | --- |
|  |  | SF | HF | SCP | LCP |
| Aggressive | Bout | 0.02±0.04 | 0.02±0.03 | 0.03±0.04 | 0.01±0.03 |
| Severe | Bout | 0.02±0.02 | 0.00±0.00 | 0.01±0.02 | 0.01±0.01 |
| Gentle | Bout | 0.09±0.06 | 0.06±0.07 | 0.04±0.05 | 0.11±0.06 |
| Peck Block | Bout | 1.11±0.46 | 1.24±0.50 | 1.29±0.57 | 1.06±0.34 |
| Peck Stone | Bout | 0.66±0.39 | 1.03±0.82 | 1.17±0.70 | 0.52±0.43 |

^1^SF = Standard Fiber; HF = High Fiber

^2^SCP = Standard Crude Protein; LCP = Low Crude Protein

Table 5: Fiber × protein and fiber × protein × age interaction effects on egg quality measured at 35 and 48 weeks of age.

| Treatment^1^ | Egg Weight (g) | Breaking Strength | Shell Thickness | Shell Weight | Yolk Color | Yolk Weight | Albumen Height | Albumen DM | Haugh Unit |
| --- | --- | --- | --- | --- | --- | --- | --- | --- | --- |
|  |  | (KgF) | (mm) | (g) |  | (g) | (mm) | (%) |  |
| Fiber × Protein | |  |  |  |  |  |  |  |  |
| SCP-SF | 64.9 | 4.66 | 0.379 | 6.57 | 11.8^b^ | 16.6 | 9.38 | 11.5 | 95.2 |
| SCP-HF | 65.1 | 4.61 | 0.379 | 6.57 | 11.9^b^ | 16.5 | 9.62 | 11.3 | 96.3 |
| LCP-SF | 64.5 | 4.82 | 0.381 | 6.58 | 12.7^a^ | 16.8 | 9.38 | 11.5 | 95.3 |
| LCP-HF | 65.5 | 4.96 | 0.380 | 6.63 | 12.0^b^ | 16.8 | 9.52 | 11.4 | 95.7 |
| SEM | 0.5 | 0.08 | 0.002 | 0.06 | 0.1 | 0.2 | 0.09 | 0.08 | 0.5 |
| Fiber × Protein × Age | | | | | | | | | |
| Week 35 |  |  |  |  |  |  |  |  |  |
| SCP-SF | 63.9 | 4.79 | 0.390 | 6.79 | 12.0 | 16.0 | 9.49 | 11.7 | 95.9 |
| SCP-HF  LCP-SF  LCP-HF | 64.9  64.0  64.4 | 4.81  4.90  5.10 | 0.387  0.392  0.392 | 6.74  6.77  6.79 | 12.2  12.7  12.1 | 15.9  16.1  16.1 | 9.89  9.76  9.68 | 11.5  11.7  11.7 | 97.6  97.2  96.7 |
| SEM | 0.5 | 0.11 | 0.003 | 0.08 | 0.1 | 0.1 | 0.10 | 0.11 | 0.5 |
| Week 48 |  |  |  |  |  |  |  |  |  |
| SCP-SF | 65.8 | 4.53 | 0.368 | 6.35 | 11.6 | 17.3 | 9.28 | 11.3 | 94.5 |
| SCP-HF  LCP-SF  LCP-HF | 65.3  65.0  66.7 | 4.42  4.73  4.81 | 0.371  0.370  0.369 | 6.39  6.39  6.46 | 11.6  12.8  11.9 | 17.1  17.4  17.6 | 9.35  8.99  9.36 | 11.0  11.2  11.2 | 95.0  93.3  94.7 |
| SEM | 0.8 | 0.07 | 0.003 | 0.07 | 0.1 | 0.3 | 0.12 | 0.1 | 0.6 |
| P Value |  |  |  |  |  |  |  |  |  |
| Fiber | 0.136 | 0.928 | 0.855 | 0.577 | 0.004 | 0.699 | 0.062 | 0.159 | 0.126 |
| Protein | 0.866 | 0.001 | 0.382 | 0.505 | <0.001 | 0.114 | 0.698 | 0.457 | 0.768 |
| Age (A) | 0.014 | <0.001 | <0.001 | <0.001 | 0.003 | <0.001 | <0.001 | <0.001 | <0.001 |
| F x P | 0.742 | 0.191 | 0.988 | 0.661 | <0.001 | 0.596 | 0.329 | 0.484 | 0.300 |
| F x A | 0.888 | 0.303 | 0.504 | 0.500 | 0.135 | 0.872 | 0.691 | 0.956 | 0.617 |
| P x A | 0.630 | 0.407 | 0.507 | 0.735 | 0.008 | 0.721 | 0.212 | 0.738 | 0.119 |
| F x P x A | 0.169 | 0.985 | 0.346 | 0.884 | 1.000 | 0.545 | 0.011^2^ | 0.603 | 0.015^2^ |

^1^SCP-SF = Standard Crude Protein and Standard Fiber (Control Diet); SCP-HF = Standard Crude Protein and High Fiber; LCP-SF= Low Crude Protein and Standard Fiber; LCP-HF = Low Crude Protein and High Fiber

^2^Within individual weeks, means did not differ significantly between treatments following the Tukey–Kramer adjustment for multiple comparisons.

Table 6: Detailed statistical outputs for production performance parameters of laying hens in response to fiber and crude protein levels from 20 to 50 weeks of age, including numerator degrees of freedom (Num df), denominator degrees of freedom (Den df), F-values, and P-values for main effects and interactions. Statistical results are presented as F(Num df, Den df) = F-value, P = P-value.

| **Effect** | **Feed Intake** | **Egg Weight** | **Hen Day Egg Production** | **Egg Mass** | **FCR** |
| --- | --- | --- | --- | --- | --- |
| Fiber | *F*(1,12) = 0.00, *P* = 0.989 | *F*(1,12) = 9.4, *P* = 0.010 | *F*(1,12) = 1.8, *P* = 0.204 | *F*(1,12) = 9.2, *P* = 0.011 | *F*(1,12) = 3.76, *P* = 0.076 |
| Protein | *F*(1,12) = 2.39, *P* = 0.148 | *F*(1,12) = 8.1, *P* = 0.015 | *F*(1,12) = 0.1, *P* = 0.807 | *F*(1,12) = 3.0, *P* = 0.109 | *F*(1,12) = 0.09, *P* = 0.773 |
| Period | *F*(7, 84) = 530.76, *P*  <0.001 | *F*(7, 84) = 2511.3, *P* <0.001 | *F*(7, 84) = 1116.9, *P* <0.001 | *F*(7, 84) = 2087.1, *P* <0.001 | *F*(7, 84) = 442.69, *P* <0.001 |
| Fiber × Protein | *F*(1, 12) = 2.75, *P* = 0.123 | *F*(1,12) = 0.3, *P* = 0.582 | *F*(1,12) = 0.6, *P* = 0.451 | *F*(1,12) = 1.6, *P* = 0.228 | *F*(1,12) = 2.18, *P* = 0.166 |
| Fiber × Period | *F*(7, 84) = 7.37, *P* <0.001 | *F*(7, 84) = 2.0, *P* = 0.069 | *F*(7, 84) = 0.6, *P* = 0.773 | *F*(7, 84) = 1.0, *P* = 0.409 | *F*(7, 84) = 7.46, *P* <0.001 |
| Protein × Period | *F*(7, 84) = 1.40, *P* = 0.216 | *F*(7, 84) = 1.4, *P* = 0.226 | *F*(7, 84) = 0.5, *P* = 0.837 | *F*(7, 84) = 0.5, *P* = 0.845 | *F*(7, 84) = 1.82, *P* = 0.093 |
| Fiber × Protein × Period | *F*(7, 84) = 4.12, *P* = 0.001 | *F*(7, 84) = 0.7, *P* = 0.678 | *F*(7, 84) = 0.4, *P* = 0.928 | *F*(7, 84) = 0.4, *P* = 0.920 | *F*(7, 84) = 2.59, *P* = 0.018 |

Table 7: Production performance of laying hens in response to fiber and crude protein levels in the diets across eight consecutive periods from 20 to 50 weeks of age, showing Fiber × Period and Protein × Period interactions.

| Factor | Level^1^ | Period^2^ | Feed Intake^3^  (g/hen/d) | Egg Weight | Hen Day Egg Production^4^ | Egg Mass | FCR^5^ |
| --- | --- | --- | --- | --- | --- | --- | --- |
|  |  | **(Weeks)** |  | **(g)** | **(%)** | **(g/hen/d)** |  |
| Fiber | SF | 20-23 | 98.3±0.73 | 51.59±0.132 | 60.0±0.74 | 31.0±0.35 | 3.18±0.045 |
|  | HF | 20-23 | 99.4±0.73 | 52.30±0.132 | 60.1±0.74 | 31.4±0.35 | 3.16±0.045 |
|  | SF | 24-27 | 114.2±0.89 | 58.53±0.128 | 97.5±0.35 | 57.1±0.20 | 2.00±0.022 |
|  | HF | 24-27 | 115.7±0.89 | 58.72±0.128 | 97.4±0.35 | 57.2±0.20 | 2.02±0.022 |
|  | SF | 28-31 | 117.7±1.66 | 61.44±0.112 | 97.7±0.35 | 60.0±0.23 | 1.96±0.029 |
|  | HF | 28-31 | 117.2±1.66 | 61.64±0.112 | 97.8±0.35 | 60.3±0.23 | 1.94±0.029 |
|  | SF | 32-35 | 121.8±1.53^a^ | 62.83±0.110 | 98.0±0.28 | 61.6±0.18 | 1.98±0.023^a^ |
|  | HF | 32-35 | 116.1±1.53^b^ | 63.03±0.110 | 98.1±0.28 | 61.9±0.18 | 1.88±0.023^b^ |
|  | SF | 36-39 | 119.2±1.45^a^ | 63.26±0.112 | 97.4±0.38 | 61.6±0.25 | 1.94±0.022^a^ |
|  | HF | 39-39 | 114.2±1.45^b^ | 63.65±0.112 | 97.9±0.38 | 62.3±0.25 | 1.83±0.022^b^ |
|  | SF | 40-43 | 129.7±1.39^a^ | 63.99±0.130 | 97.0±0.27 | 62.1±0.19 | 2.09±0.022^a^ |
|  | HF | 40-43 | 121.0±1.39^b^ | 64.39±0.130 | 97.6±0.27 | 62.9±0.19 | 1.93±0.022^b^ |
|  | SF | 44-47 | 124.0±3.19 | 64.18±0.127 | 96.4±0.49 | 61.9±0.33 | 2.00±0.046^a^ |
|  | HF | 44-47 | 115.8±3.19 | 64.72±0.127 | 97.1±0.49 | 62.8±0.33 | 1.84±0.046^b^ |
|  | SF | 48-50 | 121.2±2.49 | 64.33±0.154 | 96.1±0.47 | 61.8±0.37 | 1.96±0.034^a^ |
|  | HF | 48-50 | 113.7±2.49 | 65.00±0.154 | 97.3±0.47 | 63.2±0.37 | 1.80±0.034^b^ |
|  |  |  |  |  |  |  |  |
| Protein | SCP | 20-23 | 99.0±0.73 | 52.25±0.132 | 60.1±0.74 | 31.4±0.35 | 3.16±0.045 |
|  | LCP | 20-23 | 98.7±0.73 | 51.64±0.132 | 60.1±0.74 | 31.0±0.35 | 3.18±0.045 |
|  | SCP | 24-27 | 114.9±0.89 | 58.68±0.128 | 97.5±0.35 | 57.2±0.20 | 2.01±0.022 |
|  | LCP | 24-27 | 115.0±0.89 | 58.57±0.128 | 97.5±0.35 | 57.1±0.20 | 2.01±0.022 |
|  | SCP | 28-31 | 116.0±1.66 | 61.67±0.112 | 98.0±0.35 | 60.4±0.23 | 1.92±0.029 |
|  | LCP | 28-31 | 119.0±1.66 | 61.40±0.112 | 97.6±0.35 | 59.9±0.23 | 1.99±0.029 |
|  | SCP | 32-35 | 118.5±1.53 | 63.09±0.110 | 98.2±0.28 | 62.0±0.18 | 1.91±0.023 |
|  | LCP | 32-35 | 119.4±1.53 | 62.78±0.110 | 97.9±0.28 | 61.5±0.18 | 1.94±0.023 |
|  | SCP | 36-39 | 116.4±1.45 | 63.70±0.112 | 97.7±0.38 | 62.2±0.25 | 1.87±0.022 |
|  | LCP | 36-39 | 117.0±1.45 | 63.20±0.112 | 97.6±0.38 | 61.7±0.25 | 1.90±0.022 |
|  | SCP | 40-43 | 125.9±1.39 | 64.40±0.130 | 97.1±0.27 | 62.5±0.19 | 2.01±0.022 |
|  | LCP | 40-43 | 124.9±1.39 | 63.99±0.130 | 97.5±0.27 | 62.4±0.19 | 2.00±0.022 |
|  | SCP | 44-47 | 121.3±3.19 | 64.60±0.127 | 96.4±0.49 | 62.3±0.33 | 1.95±0.046 |
|  | LCP | 44-47 | 118.6±3.19 | 64.29±0.127 | 97.1±0.49 | 62.4±0.33 | 1.90±0.046 |
|  | SCP | 48-50 | 116.2±2.49 | 64.91±0.154 | 96.5±0.47 | 62.6±0.37 | 1.86±0.034 |
|  | LCP | 48-50 | 118.7±2.49 | 64.41±0.154 | 96.9±0.47 | 62.4±0.37 | 1.90±0.034 |
| P-Value | |  |  |  |  |  |  |
| Fiber | |  | 0.989 | 0.010 | 0.204 | 0.011 | 0.076 |
| Protein | |  | 0.148 | 0.015 | 0.807 | 0.109 | 0.773 |
| Period | |  | <0.001 | <0.001 | <0.001 | <0.001 | <0.001 |
| Fiber × Protein | |  | 0.123 | 0.582 | 0.451 | 0.228 | 0.166 |
| Fiber × Period | |  | <0.001 | 0.069 | 0.773 | 0.409 | <0.001 |
| Protein × Period | |  | 0.216 | 0.226 | 0.837 | 0.845 | 0.093 |
| Fiber × Protein × Period | |  | 0.001 | 0.678 | 0.928 | 0.920 | 0.018 |

^1^SF = Standard Fiber; HF = High Fiber; SCP = Standard Crude Protein; LCP = Low Crude Protein.

^2^Production performance parameters were measured across eight consecutive periods from 20 to 50 weeks of age (20–23, 24–27, 28–31, 32–35, 36–39, 40–43, 44–47, and 48–50 weeks of age).

^3^Feed intake was calculated on a hen-day basis, accounting for mortality and removals within each pen during the experimental period.

^4^Laying percentage was calculated on a hen-day basis using the number of hens present in each pen, accounting for mortality and removals.

^5^Feed conversion ratio calculated using hen-day adjusted feed intake and egg mass values, accounting for mortality and removals.

^a,b^Means within a period with differing superscripts differ significantly.

Table 8: Production performance of laying hens in response to dietary treatment combinations across eight consecutive periods from 20 to 50 weeks of age, showing Fiber × Protein and Fiber × Protein × Period interactions.

| Treatment^1^ | Feed Intake^2^  (g/hen/d) | | Egg Weight | Hen Day Egg Production^3^ | Egg Mass | FCR^4^ |
| --- | --- | --- | --- | --- | --- | --- |
|  |  |  | **(g)** | **(%)** | **(g/hen/d)** |  |
| Fiber × Protein | | |  |  |  |  |
| SCP-SF | 117 | | 61.50 | 92.5 | 57.3 | 2.11 |
| SCP-HF | 115 | | 61.83 | 92.8 | 57.8 | 2.06 |
| LCP-SF | 119 | | 61.04 | 92.5 | 56.9 | 2.17 |
| LCP-HF | 113 | | 61.53 | 93.0 | 57.7 | 2.04 |
| SEM | 1.95 | | 0.119 | 0.36 | 0.18 | 0.032 |
| Fiber × Protein × Period | | | |  |  |  |
| Period: 20 – 23 Weeks | | | | | | |
| SCP-SF | 97.4 | | 51.95 | 59.9 | 31.1 | 3.13 |
| SCP-HF | 100.6 | | 52.56 | 60.2 | 31.6 | 3.18 |
| LCP-SF | 99.1 | | 51.24 | 60.1 | 30.8 | 3.22 |
| LCP-HF | 98.2 | | 52.04 | 60.1 | 31.3 | 3.14 |
| SEM | 1.04 | | 0.186 | 1.05 | 0.50 | 0.064 |
| Period: 24 – 27 Weeks | | | | | | |
| SCP-SF | 112.5 | | 58.69 | 97.6 | 57.3 | 1.96 |
| SCP-HF | 117.3 | | 58.67 | 97.3 | 57.1 | 2.05 |
| LCP-SF | 116.0 | | 58.37 | 97.5 | 56.9 | 2.04 |
| LCP-HF | 114.0 | | 58.76 | 97.4 | 57.3 | 1.99 |
| SEM | 1.25 | | 0.181 | 0.50 | 0.28 | 0.031 |
| Period: 28 – 31 Weeks | | | | | | |
| SCP-SF | 114.5 | | 61.67 | 97.8 | 60.3 | 1.90 |
| SCP-HF | 117.4 | | 61.67 | 98.1 | 60.5 | 1.94 |
| LCP-SF | 120.9 | | 61.20 | 97.7 | 59.8 | 2.02 |
| LCP-HF | 117.1 | | 61.61 | 97.5 | 60.1 | 1.95 |
| SEM | 2.35 | | 0.158 | 0.50 | 0.33 | 0.041 |
| Period: 32 – 35 Weeks | | | | | | |
| SCP-SF | 121.2 | | 63.00 | 98.2 | 61.9 | 1.96 |
| SCP-HF | 115.8 | | 63.18 | 98.2 | 62.1 | 1.87 |
| LCP-SF | 122.4 | | 62.67 | 97.7 | 61.2 | 2.00 |
| LCP-HF | 116.4 | | 62.89 | 98.1 | 61.7 | 1.89 |
| SEM | 2.17 | | 0.155 | 0.40 | 0.25 | 0.032 |
| Period: 36 – 39 Weeks | | | | | | |
| SCP-SF | 120.2 | | 63.60 | 97.7 | 62.1 | 1.93 |
| SCP-HF | 112.6 | | 63.81 | 97.6 | 62.3 | 1.81 |
| LCP-SF | 118.2 | | 62.91 | 97.1 | 61.1 | 1.94 |
| LCP-HF | 115.8 | | 63.50 | 98.2 | 62.3 | 1.86 |
| SEM | 2.05 | | 0.159 | 0.54 | 0.36 | 0.031 |
| Period: 40 – 43 Weeks | | | | | | |
| SCP-SF | 130.5^a^ | | 64.22 | 97.0 | 62.3 | 2.10ᵃ |
| SCP-HF | 121.2ᵇ | | 64.57 | 97.2 | 62.8 | 1.93ᵇ |
| LCP-SF | 128.9ᵃᵇ | | 63.75 | 97.0 | 61.8 | 2.08ᵃ |
| LCP-HF | 120.8ᵇ | | 64.22 | 98.0 | 62.9 | 1.92ᵇ |
| SEM | 1.96 | | 0.183 | 0.38 | 0.27 | 0.032 |
| Period: 44 – 47 Weeks | | | | | | |
| SCP-SF | 123.5 | | 64.30 | 96.0 | 61.8 | 2.00 |
| SCP-HF | 119.0 | | 64.91 | 96.7 | 62.8 | 1.90 |
| LCP-SF | 124.5 | | 64.05 | 96.7 | 62.0 | 2.01 |
| LCP-HF | 112.6 | | 64.53 | 97.4 | 62.9 | 1.79 |
| SEM | 4.52 | | 0.180 | 0.69 | 0.47 | 0.065 |
| Period: 48 – 50 Weeks | | | | | | |
| SCP-SF | 117.5 | | 64.53 | 95.9 | 61.9 | 1.90ᵃᵇ |
| SCP-HF | 115.0 | | 65.29 | 97.0 | 63.3 | 1.82ᵇ |
| LCP-SF | 124.9 | | 64.12 | 96.2 | 61.7 | 2.02^a^ |
| LCP-HF | 112.4 | | 64.70 | 97.5 | 63.1 | 1.78ᵇ |
| SEM | 3.51 | | 0.218 | 0.66 | 0.52 | 0.048 |
| P - Value |  | |  |  |  |  |
| Fiber | | 0.989 | 0.010 | 0.204 | 0.011 | 0.076 |
| Protein | | 0.148 | 0.015 | 0.807 | 0.109 | 0.773 |
| Period | | <0.001 | <0.001 | <0.001 | <0.001 | <0.001 |
| Fiber × Protein | | 0.123 | 0.582 | 0.451 | 0.228 | 0.166 |
| Fiber × Period | | <0.001 | 0.069 | 0.773 | 0.409 | <0.001 |
| Protein × Period | | 0.216 | 0.226 | 0.837 | 0.845 | 0.093 |
| Fiber × Protein × Period | | 0.001 | 0.678 | 0.928 | 0.920 | 0.018 |

^1^SCP-SF = Standard Crude Protein and Standard Fiber (Control Diet); SCP-HF = Standard Crude Protein and High Fiber; LCP-SF= Low Crude Protein and Standard Fiber; LCP-HF = Low Crude Protein and High Fiber

^2^Feed intake was calculated on a hen-day basis, accounting for mortality and removals within each pen during the experimental period.

^3^Laying percentage was calculated on a hen-day basis using the number of hens present in each pen, accounting for mortality and removals.

^4^Feed conversion ratio calculated using hen-day adjusted feed intake and egg mass values, accounting for mortality and removals.

^a,b^Means within a period with differing superscripts differ significantly.

Table 9: Fiber × protein interaction effects on novel object test responses, expressed as the proportion of hens approaching the novel object.

| **Treatment^1^** |  | **Proportion of Hens Approaching Novel Object** |
| --- | --- | --- |
| SCP-SF |  | 0.099 |
| SCP-HF |  | 0.091 |
| LCP-SF |  | 0.090 |
| LCP-HF |  | 0.106 |
| SEM |  | 0.016 |
| P-value |  |  |
| Fiber |  | 0.881 |
| Protein |  | 0.889 |
| Age |  | 0.001 |
| Fiber × Protein | | 0.799 |
| Fiber × Age | | 0.892 |
| Protein × Age | | 0.913 |
| Fiber × Protein × Age | | 0.264 |

^1^SCP-SF = Standard Crude Protein and Standard Fiber (Control Diet); SCP-HF = Standard Crude Protein and High Fiber; LCP-SF= Low Crude Protein and Standard Fiber; LCP-HF = Low Crude Protein and High Fiber
